# The dual CCR2/CCR5 chemokine receptor antagonist Cenicriviroc reduces macrophage infiltration and disease severity in Duchenne muscular dystrophy (Dmd^mdx-4Cv^) mice

**DOI:** 10.1371/journal.pone.0194421

**Published:** 2018-03-21

**Authors:** Feng Liang, Christian Giordano, Dong Shang, Qian Li, Basil J. Petrof

**Affiliations:** 1 Meakins-Christie Laboratories and Respiratory Division, McGill University, Montreal, Quebec, Canada; 2 Program for Translational Research in Respiratory Diseases, McGill University Health Centre, Montreal, Quebec, Canada; 3 Department of Respiratory and Critical Care Medicine, First Affiliated Hospital of Xi’an JiaoTong University, Xi’an, Shaanxi, P. R. China; University of Louisville School of Medicine, UNITED STATES

## Abstract

Duchenne muscular dystrophy (DMD) is characterized by progressive muscle weakness which is ultimately fatal, most often due to involvement of the diaphragm. Macrophage infiltration of dystrophic muscles has been strongly linked to muscle damage and fibrosis in DMD. We hypothesized that cenicriviroc (CVC), a dual chemokine receptor (CCR2/CCR5) antagonist currently under clinical evaluation for other diseases, could prevent macrophage accumulation and blunt disease progression in the diaphragms of mdx mice (genetic homologue of DMD). Treatment with CVC (20 mg/kg/day intraperitoneally) or vehicle was initiated in mdx mice at 2 weeks of age (prior to the onset of muscle necrosis) and continued for 4 weeks. Flow cytometry to assess inflammatory cell subsets as well as histological and force generation parameters were determined in mdx diaphragms at the conclusion of the treatment. CVC therapy induced a major (3.9-fold) reduction in total infiltrating macrophages, whereas total numbers of neutrophils and T lymphocytes (CD4+ and CD8+) were unaffected. No changes in macrophage polarization status (inflammatory versus anti-inflammatory skewing based on iNOS and CD206 expression) were observed. Muscle fiber size and fibrosis were not altered by CVC, whereas a significant reduction in centrally nucleated fibers was found suggesting a decrease in prior necrosis-regeneration cycles. In addition, maximal isometric force production by the diaphragm was increased by CVC therapy. These results suggest that CVC or other chemokine receptor antagonists which reduce pathological macrophage infiltration may have the potential to slow disease progression in DMD.

## Introduction

Duchenne Muscular Dystrophy (DMD) is the most common X-linked lethal disorder in humans affecting up to 1 in 3500 live male births, with about a third of cases being due to new spontaneous mutations in the dystrophin gene [1]. Despite recent advances in cell- and gene-based therapies to restore dystrophin expression in affected muscles, DMD remains a devastating disease for which treatment options are non-specific and supportive. Although corticosteroids are currently the standard of care, these medications are associated with major adverse side effects including weight gain and bone fractures as well as being only transiently effective [2]. Therefore, there is an urgent need for more efficacious therapies that help to arrest DMD disease progression while also minimizing adverse side effects.

Both animal model and human data indicate that dysregulated inflammatory mechanisms play an important role in driving DMD from its earliest stages [3]. Macrophages constitute the predominant inflammatory cell type within DMD and mdx (murine homolog of DMD) muscles [4]. Monocytes originating from the bone marrow traffic to peripheral tissues, where they differentiate into macrophages that take on different phenotypic profiles including “polarization” towards inflammatory (M1) or anti-inflammatory (M2) phenotypes [5]. We recently showed a key role for the chemokine receptor CCR2 in promoting monocyte/macrophage recruitment and pathology in mdx muscles during early phases of the disease [6]. CCR2 binds to several chemokines including CCL2 (MCP-1), CCL8 (MCP-2), CCL7 (MCP-3), CCL13 (MCP-4), and CCL12 (MCP-5). These CCR2 ligands are elevated not only within the diseased muscles but also in the serum of dystrophic animals [7]. In our previous work, germline ablation of CCR2 in mdx mice improved multiple muscle parameters including force generation, and similar benefits occurred when mdx mice were treated with a CCR2-inhibiting fusion protein molecule [6]. Another chemokine receptor, CCR5, as well as its major ligands CCL3 (MIP-1α) and CCL5 (RANTES), are also highly upregulated in mdx muscles [8]. CCR5 has been implicated in monocyte recruitment [9–11] as well as in the proinflammatory polarization of macrophages [12], suggesting that it could also represent a useful therapeutic target in DMD.

Cenicriviroc (CVC) is a novel and potent small molecule antagonist of both CCR2 and CCR5, which can be administered orally on a once-daily basis in humans due to its long half-life [13–15]. Since CCR5 acts as a co-receptor for human immunodeficiency virus (HIV), CVC was initially employed in the treatment of HIV-infected individuals [16]. More recently, CVC has shown promising results in pre-clinical models of fibrofatty liver disease where it helped to limit inflammation and fibrosis [17,18], as well as in a phase 2b study of patients with non-alcoholic steatohepatitis [19]. Overall, CVC has been found to be well-tolerated and safe in over a thousand human subjects studied to date [13,19,20].

Given its high bioavailability, excellent safety profile and relative lack of side effects in human patients thus far, together with the potential roles of both CCR2 and CCR5 in promoting inflammation within dystrophic muscles, CVC is an attractive candidate drug for anti-inflammatory therapy in DMD. Accordingly, the primary goal of the current study was to test the hypothesis that treatment with CVC would be capable of mitigating disease progression in the mdx mouse model of DMD. Outcomes were focused on the diaphragm because it is the most severely affected muscle in mdx mice with respect to fibrosis and weakness [21]. Our specific objectives were to determine whether CVC treatment is capable of: 1) inhibiting proinflammatory macrophage infiltration, and 2) modifying characteristic myopathic histopathologic features and improving muscle contractility in a well established pre-clinical model of DMD.

## Methods

### Animals and drug administration

Dystrophic mdx mice (B6Ros.Cg-Dmd^mdx-4Cv^/J) were purchased from the Jackson Labs and maintained in a barrier facility unit under specific pathogen-free conditions with a 12-hour light/dark cycle and access to food and water ad libitum. The mice received a daily intraperitoneal injection with either CVC (gift from Tobira Therapeutics, Allergan; mesylate salt form) or an equal volume of its vehicle solution (10% hydroxypropyl-beta-cyclodextrin, 5% solutol HS15, pH 6). The body weight of the animals was measured regularly to maintain a daily dose of 20 mg/kg, which is based on previous studies in other inflammatory disease models [17]. The treatment was initiated at 2 weeks of age, which corresponds to the period immediately preceding the onset of skeletal muscle necrosis in mdx mice [22]. After 4 weeks of treatment, the animals were sacrificed by cervical dislocation under isoflurane anesthesia, and diaphragm muscles collected for immunologic, histologic, and physiologic investigations. All animal procedures were approved by the McGill University Animal Care and Use Committee, in accordance with the guidelines issued by the Canadian Council on Animal Care.

### Flow cytometry

Single cell suspensions were obtained from entire diaphragms (excluding the central tendon) by mincing the muscle into small pieces in ice cold PBS. The muscles were then incubated in buffered 0.2% collagenase B (Roche) solution for 1 hour at 37°C followed by filtering of the cell suspension through a 70μm cell strainer. Total viable cell numbers were first determined by Trypan blue exclusion. Cells were then resuspended in FACS buffer (PBS with 0.5% BSA), assessed for viability with Live/Dead^tm^ stain (Invitrogen), and pre-incubated in blocking solution (BD Biosciences). The cells were subsequently stained using the following fluorescently labeled antibodies: V500 labeled anti-mouse CD45 (BD Biosciences), Alexa Fluor 488 labeled anti-mouse CD11b and PE-Cy7 labeled anti-mouse F4/80 (all from BioLegend). Following staining with surface markers, cells were washed, fixed in 1% paraformaldehyde (PFA) and permeabilized using PBS/0.3% Triton. The cells were then stained intracellularly with FITC labeled anti-mouse iNOS (BD Biosciences) and APC labeled anti-mouse CD206 (BioLegend) to define prototypical “M1-like” inflammatory (iNOS+CD206-) and “M2-like” anti-inflammatory (iNOS-CD206+) macrophages as previously described [6,23,24]. Other cells were stained with FITC anti-mouse Ly6G (BioLegend) or FITC labeled anti-mouse CD3, PE-Cy7 labeled anti-mouse CD4 and PerCP labeled anti-mouse CD8 (all from BioLegend). CD45+ live cells were identified as neutrophils (CD11b+Ly6G+), macrophages (CD11b+F4/80+), and lymphocytes (CD3+, either CD4+ or CD8+). iNOS and CD206 expression were assessed on the populations generated by the gating of CD45+CD11b+F4/80+ macrophages. Appropriate FMO controls were used to set negative population gates. All data were acquired on a BD FACS Canto II. For each sample, 100,000–500,000 events were recorded. Data analysis was done using FlowJo software (Treestar Inc). Absolute numbers of cells were calculated by multiplying percentages determined from flow cytometry by the quantity of cells isolated per mg of diaphragm tissue.

### Muscle histology

Excised diaphragms were quickly frozen in liquid nitrogen-cooled 2-methylbutane (Fisher, Fairlawn, NJ) and stored at –80°C. For general morphology, cryostat sections were stained with haematoxylin and eosin (H&E) according to standard protocols. For determination of myofiber size and central nucleation, sections were fixed in 4% PFA and permeabilized in 0.1% Triton-X100. After 3 washes in PBS, the slides were incubated overnight with 1μg/ml Alexa Fluor 488 conjugate Wheat Germ Agglutinin (Molecular Probes) to allow the perimeter of individual fibers to be identified. The sections were then washed 3 times in PBS and counterstained with Hoechst 33342 (Thermofisher) to permit visualization of fiber nuclei. Microscopic images of the tissue sections were scanned using a Zeiss AxioImager M2 microscope. For quantitative computer-assisted morphometric analysis, 5 rectangular grids were placed in random fashion on the captured images. Using Fiji [25], the following histological parameters were quantified in a blinded fashion on all fibers (average of 581 ± 52 and 563 ± 44 fibers in the control vehicle and CVC groups, respectively) contained within the 5 grids: fiber cross-sectional area, Feret’s minimal diameter, the variance coefficient of muscle fiber size, and the percentage of fibers containing central nuclei. IgG staining was performed to assess myofiber sarcolemmal permeability using an anti-mouse IgG horseradish peroxidase-congugated antibody (Promega, W402B). To assess the level of ongoing muscle regeneration, immunostaining for embryonic myosin heavy chain (F1.652, Developmental Studies Hybridoma Bank) was performed. Positively staining areas for both IgG and embryonic myosin heavy chain were quantified using ImageJ and reported to the entire muscle section area as previously described [23].

### Hydroxyproline assay

Muscle collagen content was determined by quantifying hydroxyproline in muscle as previously described [23]. The muscles were homogenized in 0.5 mol/L glacial acetic acid, dried in a speed vacuum and weighed. The dried samples were hydrolyzed in 6N HCl at 110°C overnight. Following acid hydrolysis, 10μl samples were dried and resuspended in citrate-acetate buffer. Freshly prepared chloramine-T solution was added to the dried samples, and allowed to stand at room temperature for 20 minutes. Freshly prepared Ehrlich’s solution was next added and the samples were heated to 65°C for 15 minutes. The processed samples were transferred to a 96-well plate and optical densities were read at 550nm. For each assay, a standard curve was generated using known concentrations of hydroxyproline, and the sample hydroxyproline content (per mg of wet muscle) was then determined. All chemicals were purchased from Sigma.

### Evaluation of force-generating capacity

Diaphragm strips were excised and rapidly transferred into equilibrated (95% O2-5% CO2; pH 7.38) Krebs solution for measurements of isometric force production as previously described in detail [26]. After attaching the diaphragm strip to a force transducer/length servomotor system (model 300B; dual mode; Cambridge Technology), optimal length (Lo) was determined. The force-frequency relationship was measured by sequential supramaximal stimulation for 1 sec at 10, 30, 50, 100, and 150 Hz, with 2 min between each stimulation train. All muscle force data were acquired to computer at a sampling rate of 1000 Hz for later analysis. Muscle force was normalized to cross-sectional area to determine specific force, which is expressed in Newtons/cm2.

### Statistical analysis

All data are expressed as group mean values ± SE. Data were analyzed using a commercial software package (GraphPad Prism). For each parameter, values in the CVC and vehicle treatment groups were compared using Student’s t-test for independent samples. Statistical significance was set at P<0.05.

## Results

### CVC treatment reduces pathological macrophage accumulation

**Fig 1:**
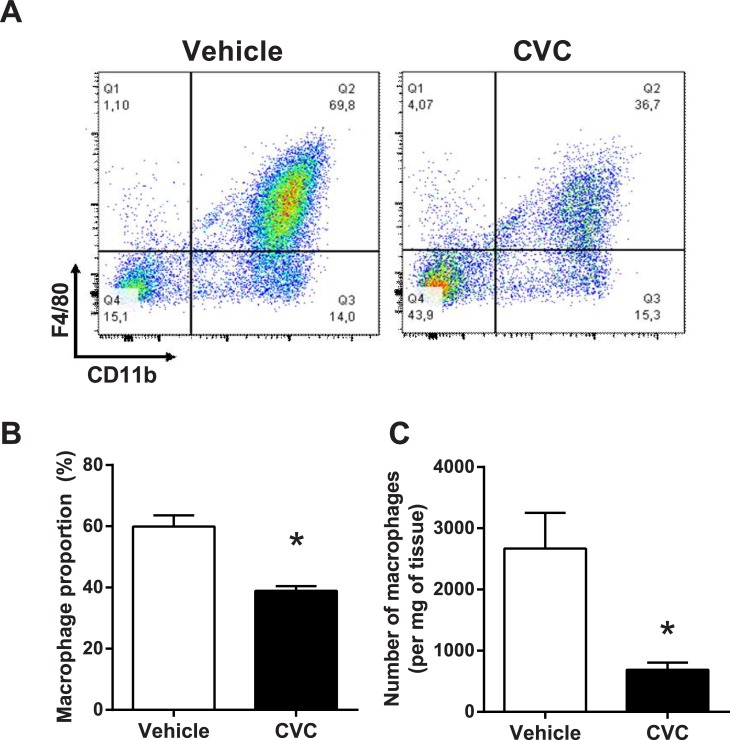
CVC effects on macrophage accumulation. (A) Representative flow cytometry plots of CD45+ leukocytes from diaphragms of vehicle- and CVC-treated mdx mice, demonstrating a marked reduction in the percentage of macrophages (CD11b+ F4/80+) present in the CVC group. (B) Group mean data (± SE) for macrophage proportion among CD45+ leukocytes in the two groups. (C) Total numbers of macrophages (normalized to muscle weight) present in mdx diaphragms after vehicle or CVC treatment. * P <0.05 for vehicle (n = 8) vs. CVC (n = 7).

Flow cytometry was performed on cell suspensions derived from the diaphragms of vehicle- and CVC-treated mice. As can be seen (Fig 1A and 1B), the proportion of macrophages present within the CD45+ leukocyte population in the dystrophic diaphragm was significantly reduced in the CVC group. This translated into a 3.9-fold decrease in the absolute number of macrophages present per mg of muscle tissue (Fig 1C).

**Fig 2:**
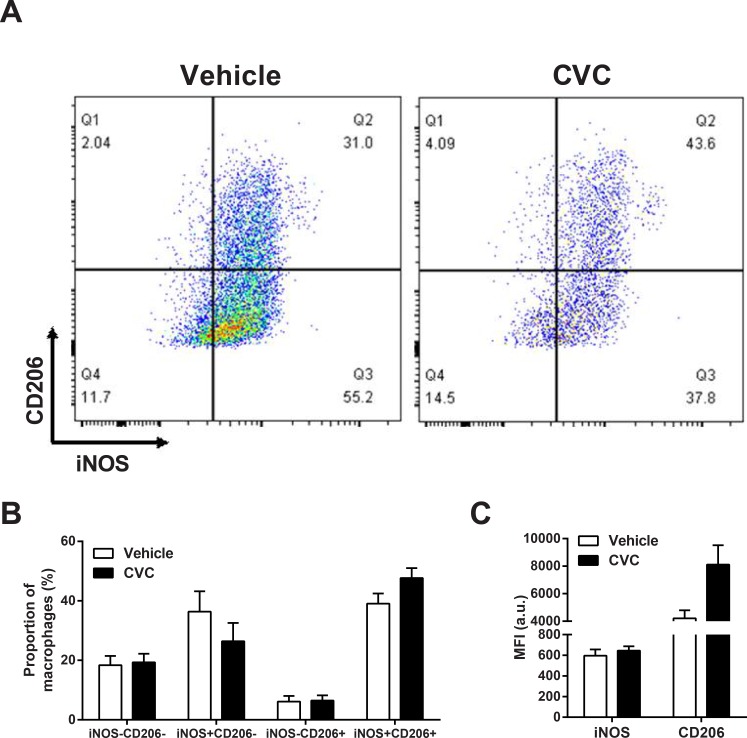
CVC effects on the macrophage polarization markers iNOS and CD206. (A) Representative flow cytometry plots of iNOS and CD206 expression on CD11b+ F4/80+ macrophages in diaphragms of vehicle- and CVC-treated mdx mice. (B) Group mean data (± SE) indicating the proportion of diaphragm macrophages showing different patterns of iNOS and CD206 expression. (C) Mean fluorescence intensity (MFI) for iNOS and CD206 expression by diaphragm macrophages in the two groups of mice. There were no statistically significant differences between the vehicle and CVC groups.

We next examined whether the balance between inflammatory (“M1-like”, defined as iNOS+ CD206-) and anti-inflammatory (“M2-like”, defined as iNOS- CD206+) macrophages, was altered by CVC treatment (Fig 2A). While there was a tendency for a lower proportion of inflammatory (iNOS+ CD206-) macrophages in the CVC group, there were no statistically significant differences in the relative proportions of the different macrophage phenotypes as defined by iNOS and/or CD206 expression (Fig 2B). In addition, the mean fluorescent intensities of iNOS and CD206 expression by intramuscular macrophages were not significantly altered although CD206 expression tended to be higher in the CVC group (Fig 2C).

**Fig 3:**
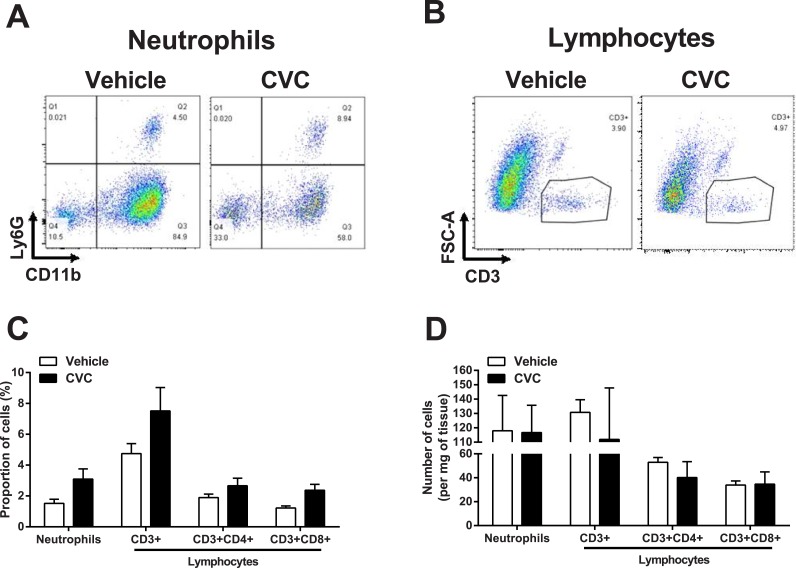
CVC effects on neutrophil and lymphocyte accumulation. Representative flow cytometry plots of (A) neutrophils (Ly6G+) and (B) lymphocytes (CD3+) in diaphragms of vehicle- and CVC-treated mdx mice. (C) Group mean data (± SE) indicating the proportion of CD45+ cells expressing characteristic neutrophil and lymphocyte markers. (D) Total numbers of neutrophils and lymphocytes (normalized to muscle weight) present in mdx diaphragms after vehicle or CVC treatment. There were no statistically significant differences between the vehicle and CVC groups.

We also determined whether CVC treatment affected neutrophil or lymphocyte populations within mdx diaphragms (Fig 3A and 3B). As expected given the large reduction in macrophages with CVC treatment, the proportions of these other leukocyte populations tended to be relatively higher in the CVC group (Fig 3C). However, absolute numbers of neutrophils and lymphocytes in the diaphragm on a per muscle weight basis were not significantly altered by CVC (Fig 3D).

### CVC effects on dystrophic disease manifestations

**Fig 4:**
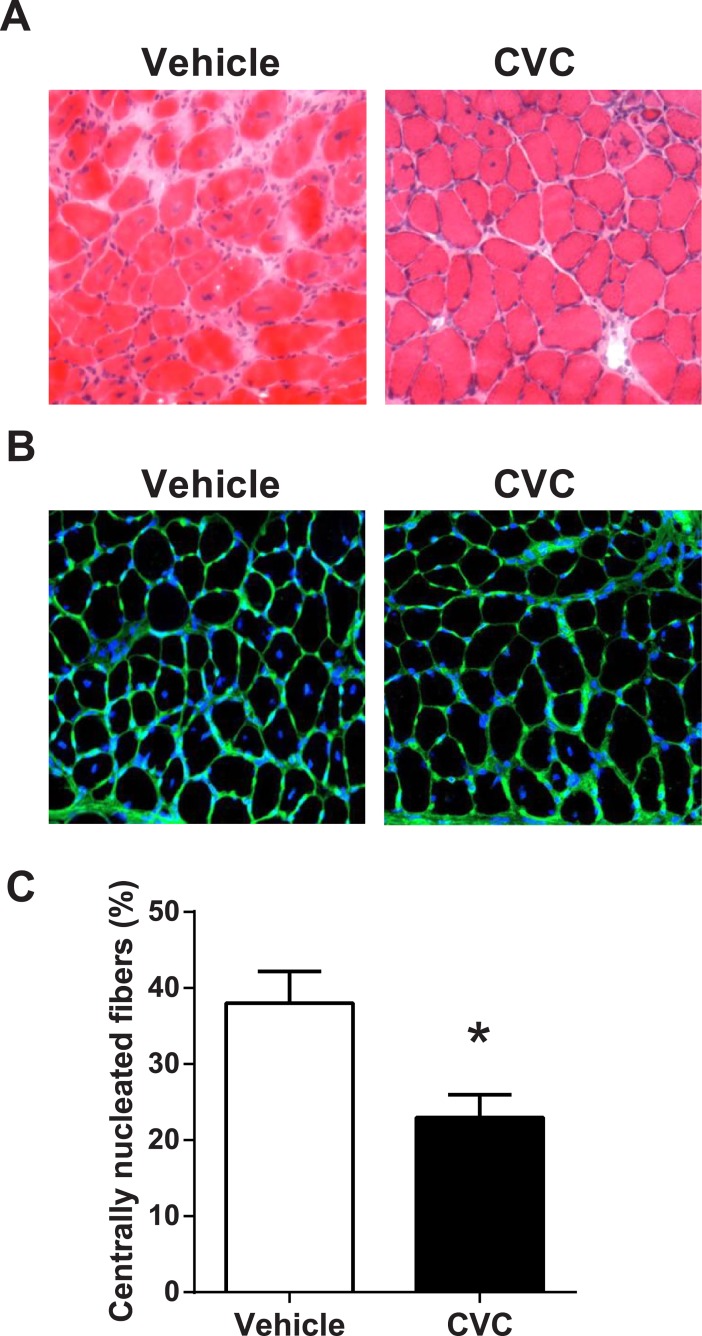
CVC effects on antecedent necrosis-regeneration cycles. Representative mdx diaphragm histological images stained with (A) Haematoxylin and eosin and (B) Agglutinin and Hoechst (blue nuclei) to reveal the location (either central or peripheral) of muscle fiber nuclei. (C) Quantification of the percentage of fibers containing centrally located nuclei, which was significantly reduced in the CVC-treated group. Values are group mean data (± SE). * P <0.05 for vehicle vs. CVC (n = 8 per group).

Treatment with CVC had no significant impact on body weight (see S1A Fig). A classical hallmark of DMD is the presence of regenerated muscle fibers containing centrally located nuclei (Fig 4A and 4B), which are considered a marker for previous necrosis-regeneration events in mdx muscles [22,27]. In the mdx mice treated with CVC, centrally nucleated fibers were significantly less prevalent than in vehicle-treated animals (Fig 4C). However, there were no differences in staining for IgG or embryonic myosin heavy chain, which reflect the current (as opposed to prior) levels of necrosis-regeneration that were present at the end of the treatment period (see S1B and S1C Fig).

**Fig 5:**
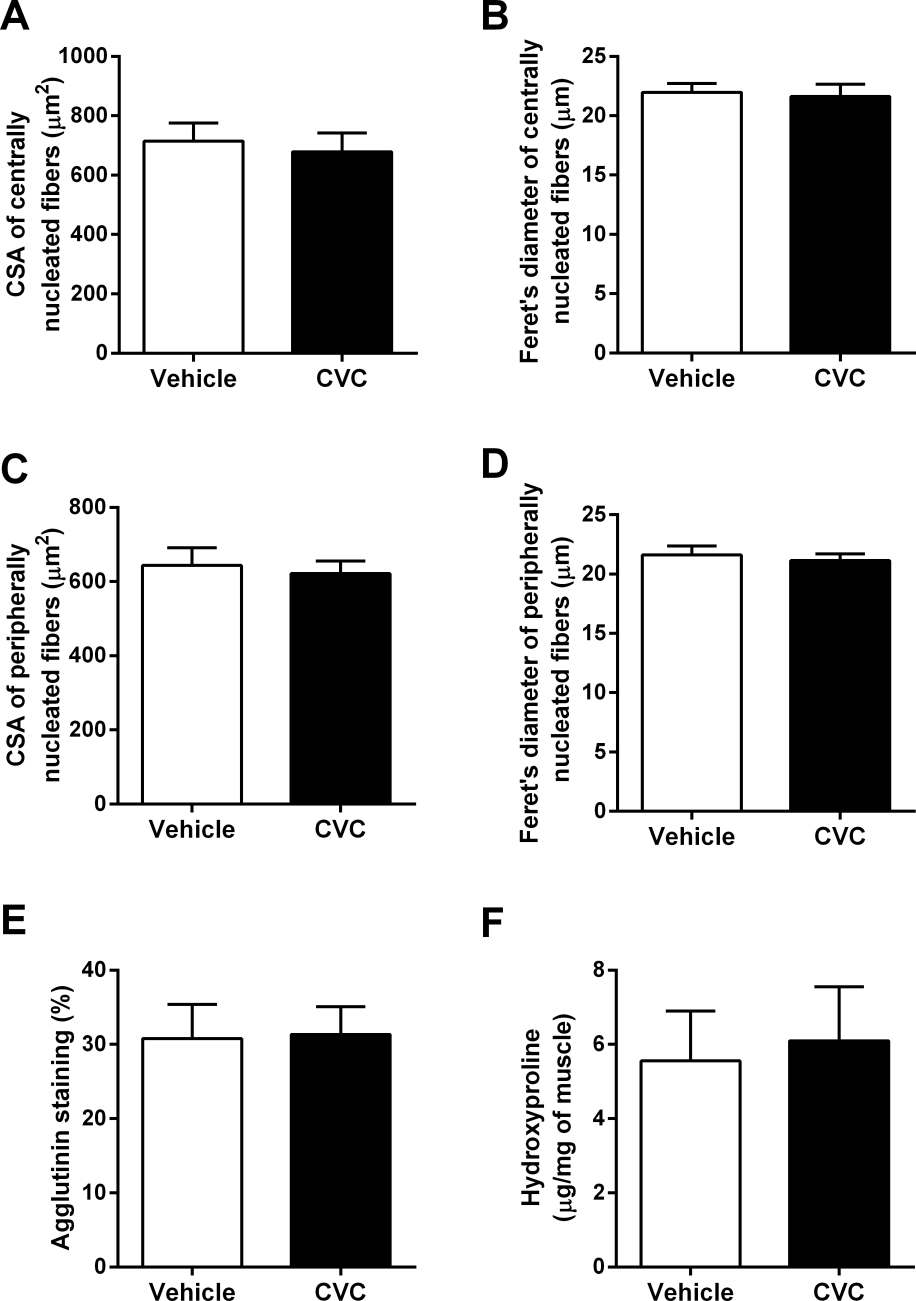
CVC effects on muscle fiber size and fibrosis. The cross-sectional area (CSA) and minimal Feret's diameter of fibers with (A, B) central and (C, D) peripheral nuclei, as well as (E) the degree of muscle fibrosis as determined by extracellular agglutinin staining, were unchanged (n = 8 mice per group for vehicle- and CVC-treated). In addition, the (F) hydroxyproline content (n = 4 mice per group) did not differ between groups. Values are group mean data (± SE).

We next evaluated the size of both centrally-nucleated and peripherally-nucleated fibers, assessed from measurements of either cross-sectional area or Feret’s minimal diameter. In centrally-nucleated (i.e., regenerated) muscle fibers, neither cross-sectional area (Fig 5A) nor Feret’s minimal diameter (Fig 5B) were affected by CVC treatment. Similarly, the size of diaphragm fibers without central nuclei (i.e., peripherally nucleated) was not altered by the CVC drug therapy (Fig 5C and 5D). The variance coefficient of fiber cross-sectional area was also unaltered by CVC (see S1D Fig).

In addition, CVC treatment did not affect either histological evidence of fibrosis (Fig 5E) or the hydroxyproline content of the muscle (Fig 5F).

**Fig 6:**
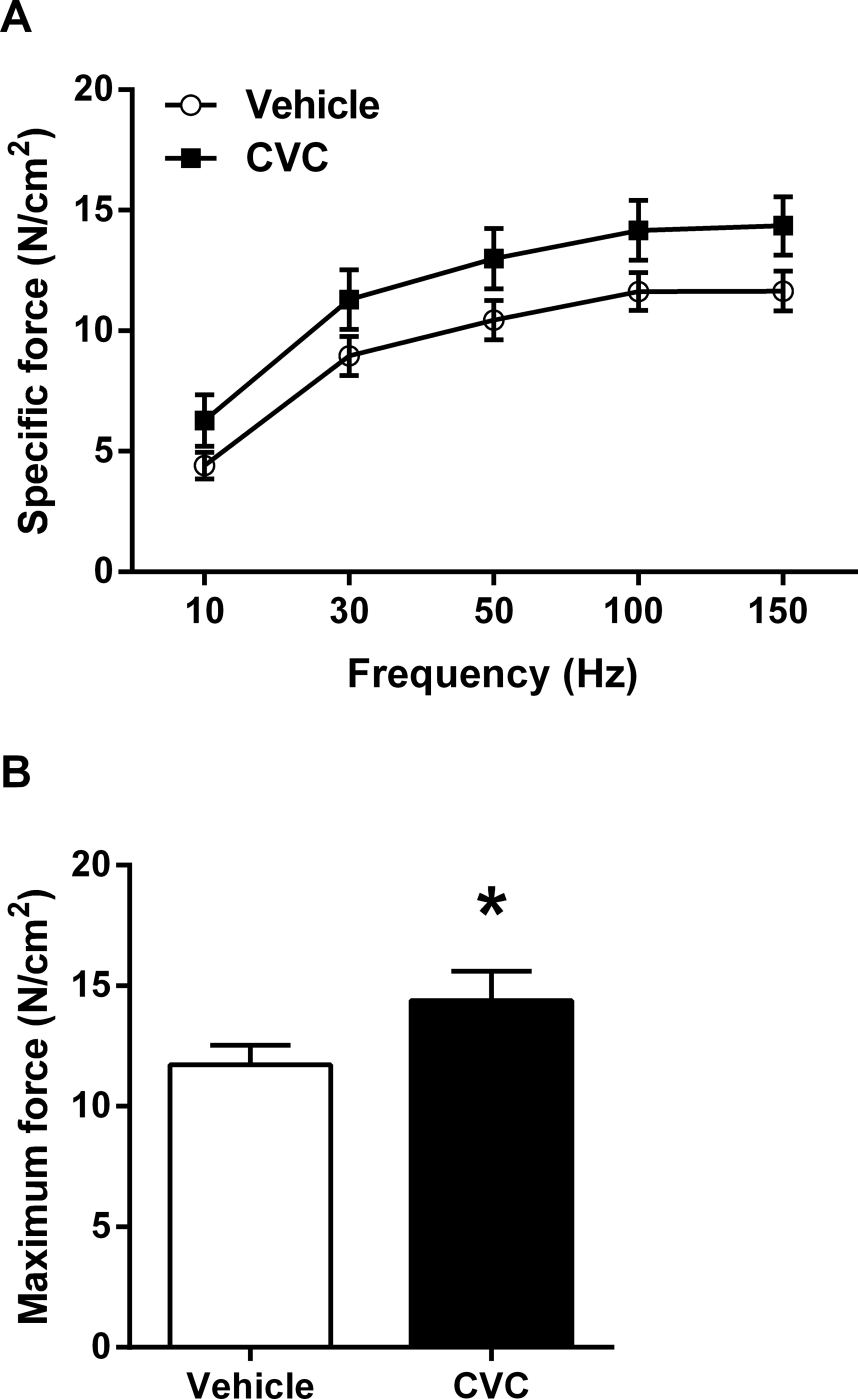
CVC effects on force generation. Ex vivo force generating capacity of the mdx diaphragm was tested (A) at different electrical stimulation frequencies, revealing (B) increased maximal isometric force generation in CVC-treated mice. Values are group mean data (± SE). * P <0.05 for vehicle vs. CVC (n = 7 per group).

Finally, the level of specific force (i.e., force normalized to muscle cross-sectional area) generated by excised diaphragm strips was determined ex vivo in the vehicle and CVC groups. Across the different electrical stimulation frequencies studied (Fig 6A), the CVC group generated higher mean values such that maximal isometric force was greater in CVC-treated mdx mice (Fig 6B).

## Discussion

Proinflammatory genes and signaling pathways are activated within DMD muscles from shortly after birth [28]. In mdx mice, blockade of the NF-kB pathway can ameliorate the disease [3,29], and several inflammatory mediators associated with innate immunity such as TNF-alpha and inducible nitric oxide synthase (iNOS) [30,31] play important roles in promoting early muscle damage. The CC class chemokines which are ligands for CCR2 and CCR5 are also expressed at abnormally high levels in DMD muscles [8,32,33]. However, to our knowledge there has been no previous evaluation of whether chemokine receptor antagonist drug therapy has any benefits for muscle disease caused by dystrophin deficiency. The main findings of the present study are that CVC, a clinically tolerable and safe CCR2/CCR5 antagonist, was able to: 1) greatly reduce macrophage infiltration in the mdx diaphragm; and 2) mitigate certain features of the disease, namely central nucleation (an indicator of antecedent necrosis) and impaired isometric force generation.

Macrophages are central players of innate immunity and act as a two-edged sword, having the ability to either promote or impede effective tissue repair. For example, in previously healthy skeletal muscles subjected to acute experimental injury, macrophage depletion delays muscle regeneration [34,35]. On the other hand, there is clear evidence that macrophages can play a deleterious role in dystrophic muscles by both exaggerating early muscle damage [4,36] and favoring the subsequent development of fibrosis [37,38]. In this regard, an important contribution of macrophages to muscle necrosis in dystrophin deficiency was first observed in macrophage depletion experiments performed in young mdx mice [4]. Later in the disease course, the elaboration of TGF-beta by infiltrating macrophages within dystrophic muscles stimulates the replacement of muscle fibers by collagen and extracelllar matrix elements [37,38].

In addition to macrophages, CVC could potentially impact upon other inflammatory cell types found within dystrophic muscles. In CCR2-deficient mice, an exaggerated neutrophilic response to skeletal muscle injury has been reported [39], and both CCR2 and CCR5 could in principle modulate lymphocyte recruitment and/or maturation. Therefore, we also determined the effects of CVC on neutrophil and lymphocyte populations in mdx diaphragms. Since macrophages normally represent the most abundant inflammatory cell type found within mdx muscles, the large reduction in total macrophage numbers induced by CVC tended to increase the relative proportions of the other leukocyte populations. However, the total numbers of neutrophils and lymphocytes adjusted for muscle weight were not modified by CVC treatment. Moreover, we did not observe any preferential skewing of the balance between CD4+ and CD8+ T lymphocytes in CVC-treated mdx mice, although we cannot entirely rule out potential effects on regulatory T cells [40,41]. Overall, the effects of CVC appeared to be largely limited to macrophages, which is similar to previous results obtained in a liver injury model [42].

In our earlier work, we demonstrated that genetic abrogation of CCR2 in mdx mice ameliorates multiple aspects of dystrophic pathology and function during early stages of disease [6]. These findings were recently confirmed by others [43]. The results of the current pharmacological study are generally consistent with the findings in CCR2-deficient mdx mice. It should be noted that a reduction in centrally nucleated fibers can in principle reflect either a decrease in prior episodes of necrosis or a failure of regeneration. The lack of greater fibrosis or weakness as well as the presence of robust embryonic myosin heavy chain staining in CVC-treated diaphragms argues against the latter possibility. However, the fact that the level of necrosis-regeneration prevailing at study termination (based on a "snapshot" of IgG and embryonic myosin staining) did not appear to differ between control and treated groups, raises the possibility that CVC benefits on myofiber pathology may have been transient and perhaps limited to the period of peak inflammation.

The effects of CVC therapy in mdx mice were for the most part less pronounced than observed with genetic deficiency of CCR2. For instance, in the genetic CCR2 deficiency model we observed an increase in the size of regenerated muscle fibers along with a reduced level of fibrosis, which was not the case for CVC-treated mdx animals. In addition, whereas the intramuscular macrophages in CCR2-deficient mdx mice showed evidence of a shift towards a more anti-inflammatory phenotype (i.e., iNOS negative, CD206 positive), a similar shift could not be unequivocally demonstrated after treatment with CVC. A lack of effect of CVC on macrophage polarization status was also reported in a recent study of liver disease [18]. We speculate that the more prominent effects of genetic CCR2 deficiency compared to CVC treatment in mdx mice could be related to a number of factors, including residual CCR2 activation in the CVC group (potentially modifiable with a higher dose or more prolonged duration of therapy), different compensatory responses to CCR2 inhibition in the two models, or the fact that CVC also acts as a CCR5 antagonist. With respect to the latter, genetic abrogation of CCR5 does not interfere with strength recovery after acute muscle injury [44], but has been recently reported to influence muscle fatigue [45]. To our knowledge, the specific role of CCR5 and its ligands in dystrophic muscle has not been explored to date.

DMD is an ultimately fatal disease for which the only widely accepted pharmacological therapy at this time is chronic corticosteroid administration, despite its very limited efficacy and substantial side effects [2]. Accordingly, there is an urgent need for new therapies. Most DMD patients die of respiratory failure due to involvement of the diaphragm, which is also the muscle in mdx mice that most closely resembles the human disease phenotype [21]. A major strength of the mdx diaphragm as an experimental model is that it exhibits early muscle fibrosis and weakness, which are only minimally present in the limb muscles of mdx mice until late in life. The fact that CVC had favorable effects in this model suggests that CVC or other small molecule chemokine antagonists could be useful as part of the therapeutic armamentarium for DMD, particularly during the early phases of the disease when macrophage infiltration is a prominent feature. Although such an approach does not correct the underlying genetic defect in DMD (i.e., dystrophin deficiency), it would nevertheless be clinically useful if secondary muscle damage caused by the innate immune system can be reduced. Moreover, it is conceivable that adjunctive chemokine inhibition strategies might also allow for the use of lower doses of corticosteroids, thereby limiting the important side effects that are frequently caused by these agents. The findings of the present investigation suggest that further studies to explore the therapeutic potential of chemokine antagonism in DMD are warranted.

## Supporting information

S1 Fig Body weight, necrosis, regenerating fibers and fiber size variability at study termination. Body weight (A, n = 7 mice per group) was determined at 6 weeks of age when mice were sacrificed for muscle physiology experiments. Fiber necrosis (B, n = 8 mice per group) and regenerating fibers (C, n = 5 mice per group) were assessed by IgG staining and embryonic myosin heavy chain staining, respectively. Variability of muscle fiber size (D, n = 8 mice per group) was evaluated by calculating the variance coefficient of fiber cross-sectional area. All above-mentioned data showed no significant differences between the control (Vehicle) and treated (CVC) groups. Values are group mean data (± SE).
(EPS)

S1 File Study data. (XLS)
